# Single-Cell ID-seq Reveals Dynamic BMP Pathway Activation Upstream of the MAF/MAFB-Program in Epidermal Differentiation

**DOI:** 10.1016/j.isci.2018.11.009

**Published:** 2018-11-09

**Authors:** Roderick A.P.M. van Eijl, Jessie A.G.L. van Buggenum, Sabine E.J. Tanis, Joost Hendriks, Klaas W. Mulder

**Affiliations:** 1 Radboud University, Faculty of Science, Radboud Institute for Molecular Life Sciences, Department of Molecular Developmental Biology, Nijmegen 6525 GA, The Netherlands

**Keywords:** Stem Cells Research, Systems Biology, Proteomics

## Abstract

Epidermal homeostasis requires balanced and coordinated adult stem cell renewal and differentiation. These processes are controlled by both extracellular signaling and by cell intrinsic transcription regulatory networks, yet how these control mechanisms are integrated to achieve this is unclear. Here, we developed single-cell Immuno-Detection by sequencing (scID-seq) and simultaneously measured 69 proteins (including 34 phosphorylated epitopes) at single-cell resolution to study the activation state of signaling pathways during human epidermal differentiation. Computational pseudo-timing inference revealed dynamic activation of the JAK-STAT, WNT, and BMP pathways along the epidermal differentiation trajectory. We found that during differentiation, cells start producing BMP2-ligands and activate the canonical intracellular effectors SMAD1/5/9. Mechanistically, the BMP pathway is responsible for activating the MAF/MAFB/ZNF750 transcription factor network to drive late-stage epidermal differentiation. Our work indicates that incorporating signaling pathway activation into this transcription regulatory network enables coordination of transcription programs during epidermal differentiation.

## Highlights

• scID-seq allows quantification of 70 (phospho-)proteins at single-cell level
• Pseudo-time inference reveals signaling dynamics during epidermal differentiation
• BMP signaling drives a late differentiation transcription program
• BMP signaling activates the MAF/MAFB/ZNF750 transcription factor network

## Graphical Abstract

**Figure:**
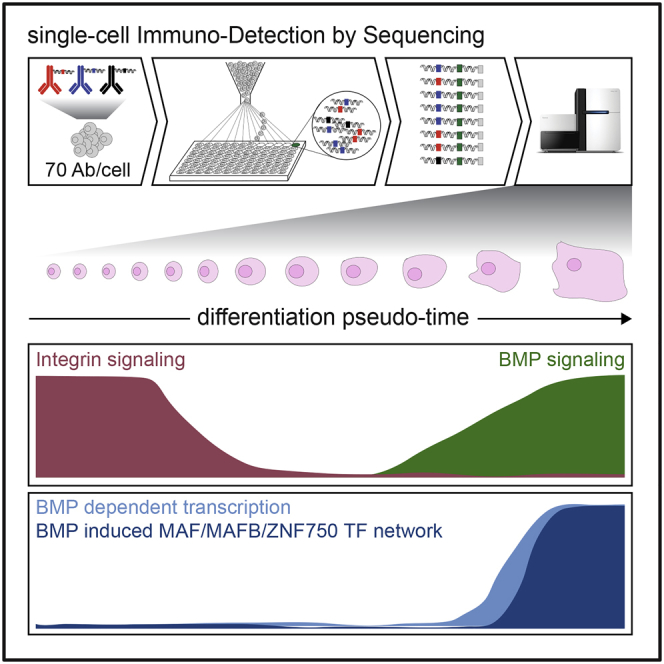

## Introduction

The human epidermis is continuously turned over throughout life, a process that requires precise control and coordination of stem cell renewal and differentiation. During epidermal homeostasis, proliferating stem/progenitor cells residing in the basal layer replenish terminally differentiated cells that are shed from the skin surface (Blanpain and Fuchs, 2006, Solanas and Benitah, 2013, Watt et al., 2006). Human epidermal stem cells can be maintained in culture and used to regenerate functional epidermis *in vivo* upon transplantation and retain their capacity to differentiate *in vitro* (Barrandon et al., 2012, Green, 2008, Hirsch et al., 2017, Rheinwald and Green, 1975). The process of differentiation is driven forward by consecutive activation of transcriptional programs, yet the mechanisms underlying their sequence and timing are not well understood. In the epidermal basal layer, cells receive proliferative signals, predominantly via the epidermal growth factor receptor, and contact the underlying basement membrane (Watt, 2002, Watt and Huck, 2013). These contacts are mediated by focal adhesions and hemi-desmosomes that contain integrin β1 and integrin α6, respectively (Watt, 2002, Watt and Huck, 2013). As cells stop proliferating and initiate differentiation, these structures are resolved, allowing the cells to detach from the basement membrane and migrate up toward the epidermal surface. During this migration, the cells undergo major transitions in transcriptional programs, eventually producing the terminally differentiated keratinocytes that form the outermost protective layer of our skin.

TP63 is a key DNA-binding transcription factor in epidermal stem cell renewal and, upon differentiation, its expression is decreased (Kouwenhoven et al., 2010, Truong et al., 2006). In contrast, other transcription factors, including KLF4, OVOL2, GRHL3, MAF/MAFB, and ZNF750, are induced (Bhaduri et al., 2015, Koster and Roop, 2004, Lopez-Pajares et al., 2015, Sen et al., 2012, Wells et al., 2009). Of these, MAF and MAFB cooperatively regulate a transcription program that includes ZNF750, which subsequently drives expression of terminal differentiation genes (Lopez-Pajares et al., 2015). This concept of sequential activation of transcription factors (also called transcription regulatory networks) explains cell intrinsic progression of epidermal differentiation. Indeed, human keratinocytes differentiate when placed in conditions where they are not in contact with other cells, for instance, in suspension in methylcellulose or on micro-patterned islands (Adams and Watt, 1989, Connelly et al., 2010). However, this does not take into account the need for regional coordination of differentiation in a tissue context. For instance, the basal, spinous, granular, and cornified layers of the epidermis are morphologically distinct and can be distinguished using specific markers, reflecting differences in transcriptional programs. The fact that these are sequentially formed layers indicates the need for a form of coordination that is not immediately explained by the function of cell intrinsic transcription factor networks.

Extracellular signaling pathways generally depend on binding of a peptide ligand to the extracellular part of a transmembrane receptor. This receptor then relays this signal into an intracellular cascade, usually involving multiple kinases and phosphorylation events, to regulate specific transcription programs. As such, activation of extracellular signaling pathways may serve as a self-contained timing mechanism to drive differentiation forward in a tissue and safeguard the irreversibility of the process. Several signaling pathways (e.g., Integrins, EGF, TGFβ, Notch, and BMP) have been implicated in epidermal biology, yet their temporal dynamics and mechanistic contributions to the control of specific transcription programs are largely unknown, especially in the context of human epidermis (Beck and Blanpain, 2012, Blanpain and Fuchs, 2006, Li et al., 2003, Watt, 2002, Watt et al., 2006). For example, the importance of the Bone Morphogenetic Protein (BMP) pathway in the embryonic morphogenesis of mouse hair follicles and bulge stem cell behavior during postnatal hair differentiation and cycling in mice is well established (Blanpain and Fuchs, 2006, Botchkarev and Sharov, 2004, Guha et al., 2004, Lewis et al., 2014, Mou et al., 2016), yet the contribution of this pathway to human epidermal renewal and differentiation is still poorly understood (Fessing et al., 2010, Gosselet et al., 2007, Yang et al., 2006).

To study the role of signaling in human epidermal differentiation, we recently described the Immuno-Detection by sequencing (ID-seq) technology to simultaneously quantify >70 (phospho-)proteins in many cell populations in parallel (van Buggenum et al., 2016, van Buggenum et al., 2018). This allowed us to screen hundreds of small molecule kinase inhibitors for their impact on keratinocyte biology. The ID-seq technology entails highly multiplexed immuno-staining with DNA-barcoded antibodies followed by signal quantification through high-throughput sequencing. A 10-nt antibody-specific barcode enables deconvolution of the measured epitope, whereas inclusion of unique molecular identifiers allows accurate count-based quantification. The fact that these 10-nt barcodes are discrete and specific for each antibody facilitates multiplexed detection of 70 (or more) epitopes per treated population of cells. Thus, using ID-seq, the activity of a range of processes and signaling pathways can be monitored through the activated (phosphorylated) states of its components (van Buggenum et al., 2018). However, populations of cells rarely display homogeneous timing of differentiation (Altschuler and Wu, 2010, Wu and Singh, 2012), making it difficult to investigate the signaling dynamics underlying this process. We therefore set out to study signaling pathway activity and dynamics during human epidermal cell differentiation at the single-cell level.

## Results

### scID-seq Allows Robust, Reproducible, and Highly Multiplexed Protein Detection in Single Cells

**Figure 1:**
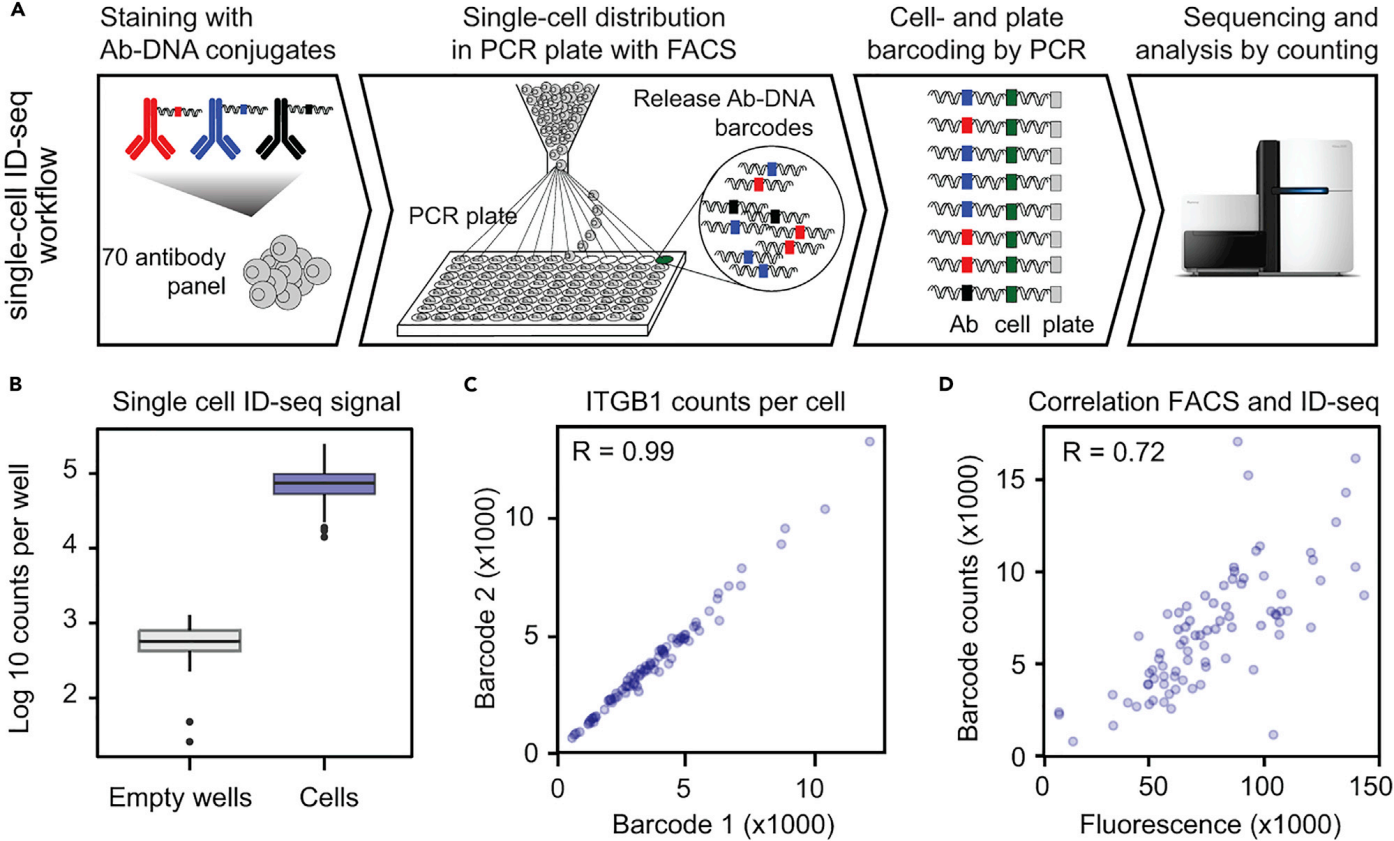
Single-Cell Immuno-Detection by Sequencing (scID-seq) Enables Robust and Reproducible Protein Detection in Individual Cells (A) Schematic overview of the scID-seq workflow. (B) Single-cell-derived antibody counts indicate low technical noise levels in scID-seq. Single cells stained with antibody-DNA conjugates sorted into individual wells of a 96-well plate and compared with wells where no cells were sorted into using scID-seq (n = 132 with 84 cells and 48 empty wells). (C) scID-seq reproducibly measures antibody signals in single cells. Independently generated antibody-DNA conjugates were used to stain human epidermal stem cells. Barcode counts derived from the two barcodes were plotted against each other, indicating high reproducibility of the protein measurements from the same cell. (D) scID-seq reflects fluorescent activated cell sorting measurements. ITGB1 levels were measured in human epidermal stem cells using FACS and scID-seq. Fluorescent signal and scID-seq counts for each cell (n = 84) showed a good correlation (R = 0.76).

To reach single-cell sensitivity and resolution, we redesigned and significantly modified our original ID-seq technology. The final single-cell (sc) ID-seq workflow includes immuno-staining of cells in suspension followed by flow-cytometry-based single-cell distribution into 96-well PCR plates to prepare samples for sequencing (Figures 1A and S1). A key improvement was the addition of a preamplification step before adding the cell-specific barcode. Again, the inclusion of a 15-nt unique molecular identifier (UMI) allowed counting-based quantification and ensured that potential duplication artifacts introduced during sample preparation could be accounted for (Kivioja et al., 2012). We found that unique barcode counts from wells containing a single sorted cell were 100-fold higher than from empty wells, indicating that only 1% of the signal constituted technical background (Figure 1B). Next, we sought to characterize the reproducibility/confidence of quantification of antibody-derived UMI counts within single cells. For this, we generated a panel of five antibodies against cell surface (ITGA6 and ITGB1), cytoplasmic (Actin and TGM1), and nuclear (RNApol2) epitopes, each of which was independently conjugated to nine distinct DNA barcodes, generating a total pool of 45 antibody-DNA conjugates. Each of these barcodes serves as a technical replicate, and their concordance therefore reflects the reproducibility of single-cell ID-seq measurements. All of the barcodes for each of these five antibodies showed very good correlation across cells (R = 0.95–0.99), indicating a low level of noise in scID-seq measurements (Figures 1C and S2). To further validate scID-seq, we stained human epidermal stem cells simultaneously with ITGB1 antibodies containing either a fluorescent group or the DNA barcode. We recorded the FACS-based ITGB1 signal, as well as the ITGB1 DNA-barcode counts for individual cells. This revealed that scID-seq counts indeed reflect standard FACS measurements for the same cell (R = 0.76, Figure 1D). Together, these results establish single-cell ID-seq as a robust and reproducible method to measure proteins at individual cell resolution. Moreover, the fact that each DNA barcode is unique to a specific antibody allows antibodies to be multiplexed and measured in each individual cell at an unprecedented level.

### scID-seq Distinguishes Renewing and Differentiated Epidermal Cells

We applied single-cell ID-seq to monitor the activity of signaling pathways and other cellular processes during epidermal differentiation using a panel of 70 antibody-DNA conjugates (van Buggenum et al., 2016, van Buggenum et al., 2018). These antibodies cover a broad range of cellular processes, including cell cycle, DNA damage, epidermal self-renewal, and differentiation, as well as the intracellular signaling status for the EGF, G-protein-coupled receptors, calcium signaling, TNFα, TGFβ, Notch, WNT, and BMP pathways (van Buggenum et al., 2018 and Table S1). This panel includes 34 antibodies against phosphorylated epitopes and was extensively validated (van Buggenum et al., 2018). For 11 of the phosphorylated epitopes we also measured the non-phosphorylated protein, allowing us to correct for total protein levels by calculating the phosphorylated to total protein ratio per cell. Most of the targeted processes are measured using multiple (three to five) independent validated antibodies (van Buggenum et al., 2018).

**Figure 2:**
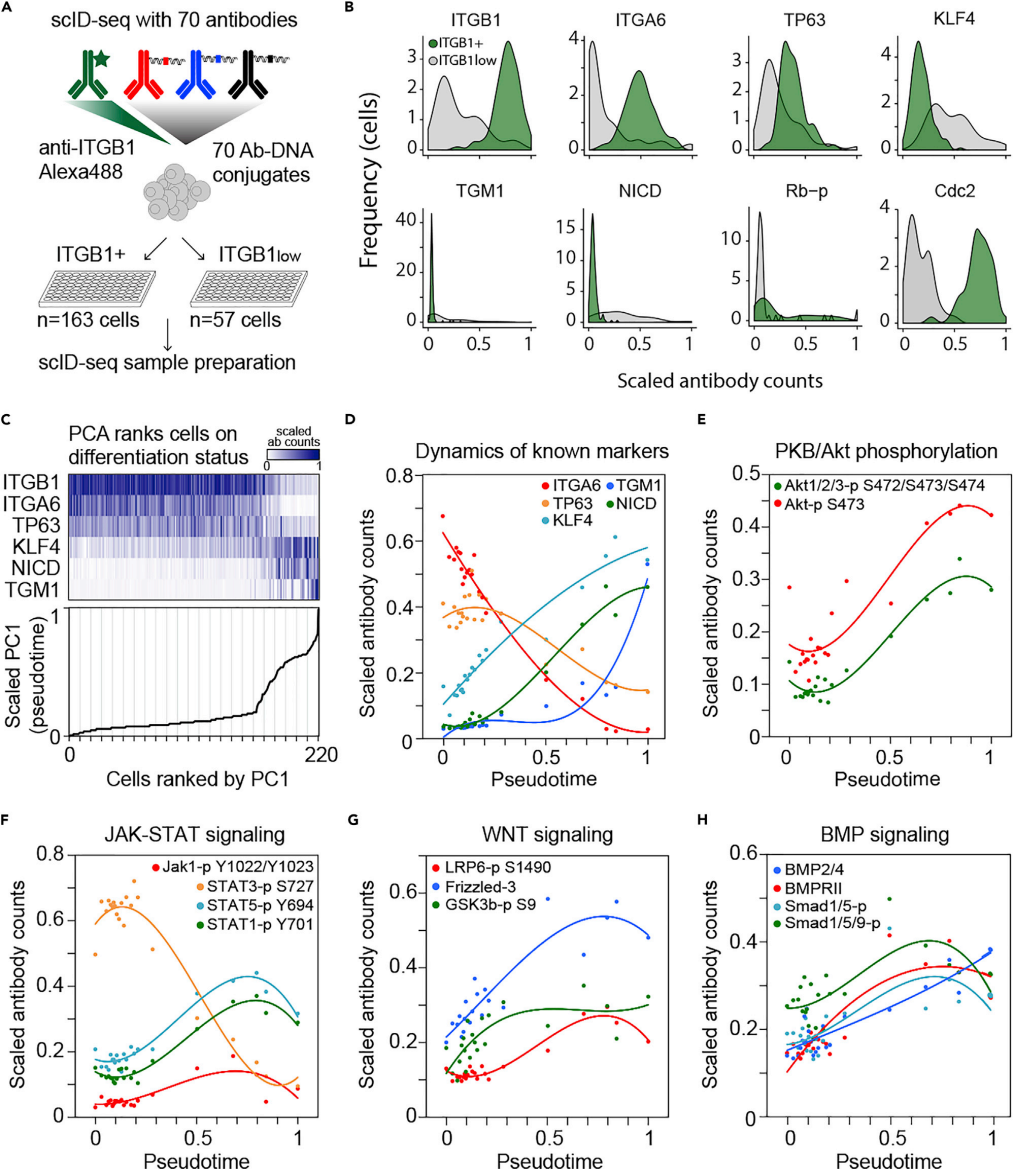
Pseudo-Timing Reveals Dynamic Signaling Pathway Activity over the Course of Epidermal Differentiation (A) Combining scID-seq with FACS-based sorting on ITGB1 levels. Cells were immuno-stained with fluorescent ITGB1 antibodies in combination with a panel of 70 Ab-DNA conjugates, FACS sorted based on their ITGB1 levels and subjected to scID-seq. (B) scID-seq distinguishes ITGB1+ and ITGB1low sorted cells based on known epidermal basal, differentiation, and cell-cycle markers. Distributions of normalized and scaled scID-seq counts of selected proteins verified the separation of the ITGB1+ and ITGB1low populations. (C) Principal-component analysis on known markers orders epidermal cells on their renewal and differentiation status. Top panel: markers used for the temporal ordering, color intensity represents scaled antibody counts. Bottom panel: cells were ranked on their (scaled) PC1 loading. Vertical lines indicate the 10-cell bins used to smoothen the data in subsequent analyses. (D) Dynamics of the markers used for PCA, ordered by pseudo-time (scaled PC1 loading) after smoothening. Data points indicate 10-cell bin mean, and solid lines represent model fit of the data (third-order polynomial regression). (E) Dynamics of two independent phosphorylated Akt/PKB antibodies over pseudo-time. (F–H) Dynamics of antibodies reflecting the JAK-STAT, WNT, and BMP signaling pathways over pseudo-time.

The surface level of ITGB1 reflects a cell's potential to proliferate and self-renew (Adams and Watt, 1989, Jones and Watt, 1993, Jones et al., 1995, Watt, 2002, Watt and Jones, 1993). We used FACS isolation of single cells based on their ITGB1 levels to capture the transitions that underlie the differentiation process (Figure 2A, see Figure S3 for details of the FACS-isolation). Colony formation assays confirmed the loss of proliferative capacity in cells expressing low levels of ITGB1 (Figure S4A). After staining with the 70 antibody-DNA conjugates, cells were sorted into ITGB1-positive (ITGB+) and ITGB1low populations based on their fluorescent ITGB1 antibody signal and subjected to scID-seq. After quality control and filtering (see methods for details), we obtained a dataset of 220 single cells (163 ITGB1+ and 57 ITGB1low, respectively) in which 69 antibody-DNA conjugates were quantified. For each cell, antibody-DNA counts were normalized for differences in sequencing depth by subsampling. To avoid downstream analyses from being dominated by the most abundant epitopes, we scaled all antibody counts between 0 and 1 across all cells. Unsupervised principal-component analysis (PCA) separated the ITGB1low (differentiated) cells from the ITGB1+ cells, confirming the notion that these represent distinct cell states (Figure S4B). Although we isolated cells only with respect to their cell surface expression of ITGB1, we found that other proteins displayed concordant dynamics. For instance, the basal cell markers ITGA6 and TP63 were also decreased in the ITGB1low population (Figure 2B). In contrast, this population exhibited higher expression of the differentiation-associated proteins TGM1, Notch1 Intracellular Domain (NICD), and KLF4 (Figure 2B). Furthermore, cell cycle markers, such as Rb-p and cdc2 (reflecting the G1-S and G2-M transitions, respectively), distinguished these populations very well (Figure 2B). This is consistent with the loss of proliferative capacity during differentiation as observed in our colony formation assay (Figure S4A) and demonstrates that scID-seq captures molecular events underlying cellular function. Indeed, other antibodies in our panel also showed quantitative differences between these two cell states in either all or in a subset of the cells (Figure S4C). Interestingly, many of the measured proteins displayed multimodal distributions, suggesting underlying subpopulation structures that could reflect transitions over the course of differentiation. This highlights the richness and complexity of our dataset and indicates the need to incorporate information on multiple parameters simultaneously in further analyses.

### Pseudo-timing Reveals Dynamic Signaling Pathway Activity over the Course of Differentiation

To obtain insight into the progressive changes in signaling pathway activity that occur over the course of epidermal cell differentiation, we aimed to order the cells by their relative differentiation state. We hypothesized that this state can be inferred by examining the combination of expression of known basal and differentiation markers. We performed PCA on selected markers (ITGB1, ITGA6, TP63, NICD, KLF4, and TGM1) with established roles and dynamics during epidermal differentiation and ranked the cells based on the resulting principal components. This revealed that principal component 1 (PC1; explaining 50% of the variance) recapitulated the expected trajectory of the epidermal differentiation process (Figure 2C). We calculated the average of 10 cell bins to smoothen the data, revealing that the basal markers ITGA6 and TP63 are indeed downregulated with distinct kinetics (Figure 2D). Immunohistochemical analysis of human epidermis showed that this PCA-derived ordering of the cells closely resembles the relative order and timing of changes of these markers *in vivo* (Figure S5). Thus, we interpret the scaled and binned PC1-score as a “pseudo-time” approximation of epidermal differentiation dynamics (Figure 2D).

Next, we mined our data for signaling pathways of which the included antibodies showed concordant effects over pseudo-time and were statistically significantly different between the ITGB1low and ITGB1+ (Figure S4C, Kolmogorov-Smirnov [K-S] test, p < 0.001). This uncovered several signaling pathways that displayed dynamic behavior over pseudo-time. For instance, PKB/Akt phosphorylation, measured by two independent antibodies, was increased upon differentiation as previously described (Figure 2E; Janes et al., 2009). Besides these expected effects, we found previously unappreciated dynamics in three additional pathways. The JAK-STAT pathway was activated during differentiation, as evident from increased phosphorylation of JAK1, STAT1, and STAT5, but not the epidermal growth factor receptor (EGFR)-activated STAT3 (Figure 2F). In addition, the level of the WNT receptor Frizzled-3 gradually increased with differentiation, as did the activated/phosphorylated form of its co-receptor LRP6 (Figure 2G). Moreover, the inactivating serine-9 phosphorylation of GSK3-β increased and then reached a plateau. This modification helps stabilize cytoplasmic β-catenin in response to WNT-pathway activation (van Kappel and Maurice, 2017). Finally, the BMP pathway was activated at multiple levels (Figure 2H). Our scID-seq panel included antibodies for the BMP2/4 ligand, the type 2 BMP receptor, total SMAD1 levels, as well as two distinct antibodies against phosphorylated SMAD1/5/9. Over pseudo-time we observed increasing levels of the ligand, the receptor, as well as phosphorylated SMADs, reflecting activation of the BMP pathway during differentiation (Figure 2H). As our cells are cultured in a defined medium in the absence of feeder cells, the signals that activate these pathways are therefore likely to be generated by the cells themselves. Indeed, the increase of the BMP2/4 ligand is consistent with such regulation by the BMP pathway (Figure 2H).

### BMP Signaling Stimulates a Terminal Epidermal Differentiation Transcription Program

**Figure 3:**
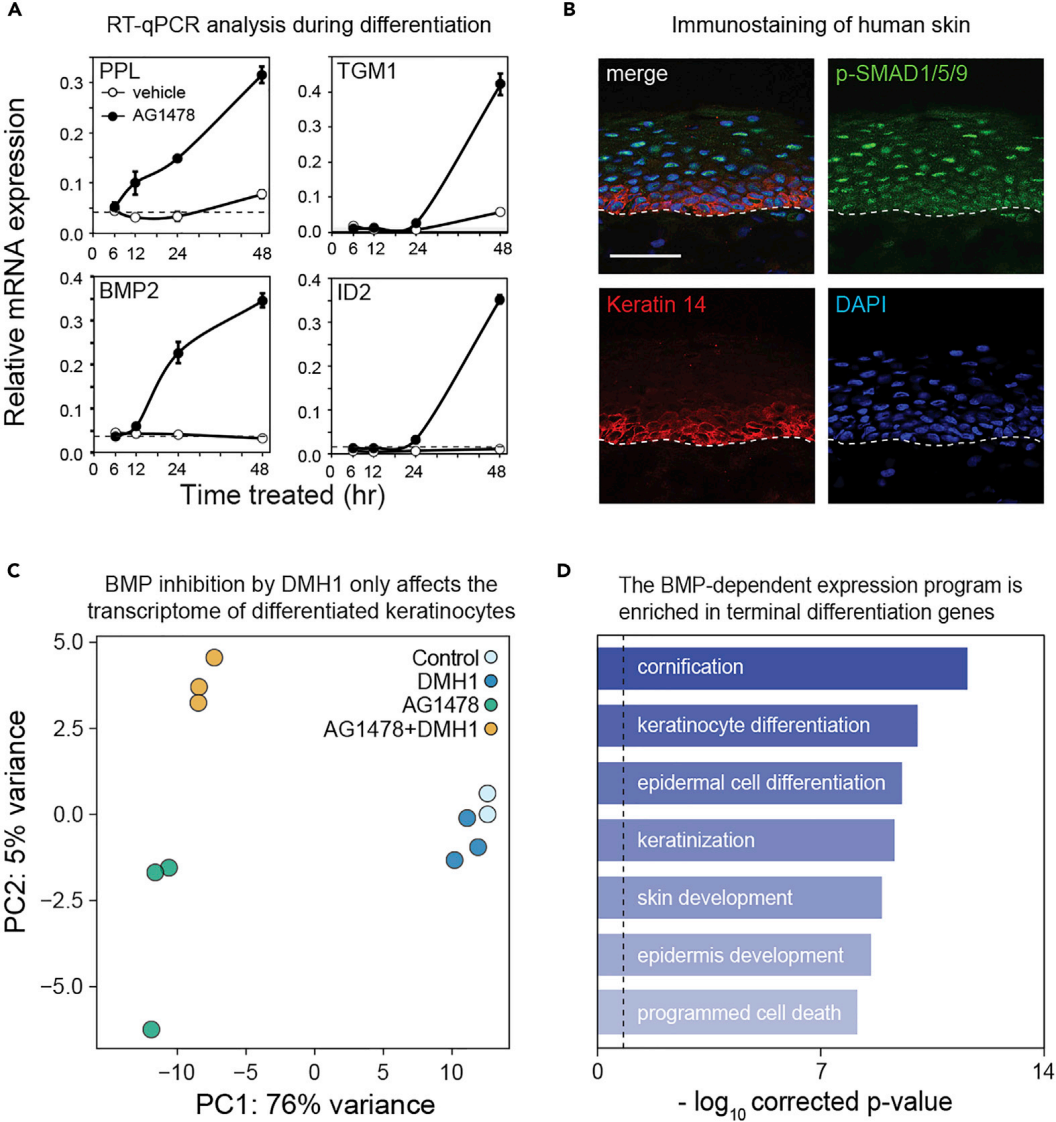
BMP Signaling Stimulates a Terminal Epidermal Differentiation Transcription Program (A) BMP2 ligand and its downstream target gene ID2 are activated during *in vitro* keratinocyte differentiation. Human keratinocytes were induced to differentiate with AG1478 (or DMSO as a control), and samples were harvested at the indicated time points. RT-qPCR analysis was performed for PPL, TGM1, BMP2, and ID2 (n = 3, data represented as mean ± SD). (B) BMP signaling is activated during epidermal differentiation *in vivo*. Sections of human foreskin were stained with antibodies against phosphorylated SMAD1/5/9. DAPI and K14 antibodies were used to counterstain all nuclei and basal cells, respectively. Scale bar represents 50 μm. Dashed line indicates the basement membrane separating the dermis and epidermis. (C) Activation of BMP signaling regulates a transcriptional program during epidermal differentiation. Principal-component analysis of human keratinocytes induced to differentiate with AG1478 (or the DMSO control) for 96 hr in the presence of the BMP receptor inhibitor DMH1, followed by RNA sequencing analysis (n = 3, except DMSO control n = 2). (D) BMP-dependent genes are involved in late differentiation processes. Top enriched terms from a gene ontology overrepresentation analysis (hypergeometric test, FDR<0.01) of differentially expressed genes between the AG1478 and AG1478+DMH1 samples.

To validate our findings on the activation of the BMP pathway, we induced differentiation of proliferating epidermal cells in culture by inhibiting EGF signaling (Kolev et al., 2008, Mulder et al., 2012). Samples were collected at 6, 12, 24, and 48 hr after the addition of either vehicle (DMSO) or the EGFR inhibitor AG1478. RT-qPCR analysis showed that mRNA expression of the early differentiation marker periplakin (PPL) and the late differentiation marker TGM1 reflected the progression of differentiation over time (Figure 3A). In line with our scID-seq results, mRNA expression of the BMP2 ligand was activated upon induction of differentiation (after the 12-hr time point), whereas the classical BMP-pathway target gene ID2 was induced at a subsequent stage (after 24 hr, Figure 3A). This induction was dependent on both BMP-ligand binding to the extracellular part of the receptor and intracellular BMPR kinase activity, as a recombinant version of the natural BMP-antagonist noggin and the small molecule kinase inhibitor DMH1 blocked ID2 expression (Figures S6A and S6B). Moreover, the observed induction of BMP2 and ID2 was further validated in cells undergoing calcium-induced differentiation (Figure S6C, data from Kretz et al., 2012). Thus, the BMP pathway is indeed activated during epidermal differentiation *in vitro*. To investigate BMP pathway activity *in vivo*, we performed immuno-staining with antibodies against phosphorylated SMAD1/5/9 on human skin. The epidermal basal layer was visualized through co-staining with a keratin-14 antibody, and we counterstained the DNA of all cells with DAPI (Figure 3B). Consistent with our scID-seq and RT-qPCR results, the signal from the p-SMAD1/5/9 antibody was increased in the differentiated (keratin-14 negative) layers of human epidermis, confirming that BMP signaling is activated during differentiation *in vivo*.

These results suggest that BMP signaling may serve as a positive feedforward loop to stimulate epidermal differentiation gene expression. To test this hypothesis, we treated cells with AG1478 for 96 hr in the absence or presence of the small molecule BMP receptor inhibitor DMH1 and monitored global gene expression by RNA sequencing. Vehicle-treated cells ± DMH1 were included as controls. PCA indicated that the transcriptomes of non-differentiated and differentiated cells were markedly different (Figure 3C, PC1; explaining 76% of the variance). In addition, PC2 (5% of variance) distinguished the DMH1 treated and non-treated differentiated cells, reflecting a transcription program that depends on the BMP pathway (Figure 3C). Notably, vehicle control cells were virtually indistinguishable from control cells treated with DMH1, indicating that BMP signaling specifically regulates differentiation but not proliferation/renewal programs. Moreover, the genes that were dependent on the BMP pathway activity showed highly significant enrichment of genes involved epidermal keratinization and cornification, indicating that BMP signaling drives transcriptional changes toward terminal differentiation (Figure 3D).

### The Terminal Differentiation Transcription Factors MAF/MAFB Are Downstream Targets of the BMP Pathway

We further explored the mechanistic role of the BMP pathway in epidermal differentiation by investigating the gene expression program that is influenced by stimulation with recombinant BMPs. First, we treated cells with different BMP ligands and measured the induction of the late differentiation marker transglutaminase I (TGM1) at the protein level. This indicated that most recombinant ligands led to a robust increase of TGM1 (Figure S7A). Moreover, simultaneous treatment of cells with the EGFR inhibitor AG1478 and BMP ligands resulted in a synergistic increase of TGM1 protein levels, highlighting opposing roles for these pathways in epidermal biology (Figure S7B). Next, we performed RNA sequencing analysis on cultured human epidermal cells treated with vehicle, AG1478, BMP2/7, or AG1478 + BMP2/7 for 48 hr (Figures S7C and S7D). Comparing the mRNA profiles of these conditions revealed the genes that were responsive to the addition of recombinant BMP2/7 and were also dynamically expressed during AG1478-induced differentiation (Figure S7E). As expected, the top most BMP-responsive genes included the ID gene family. Moreover, genes involved in terminal differentiation, including TGM1, MAF, MAFB, and ZNF750, displayed BMP-induced expression changes (Figure S7F), confirming the results obtained with the BMP pathway inhibitor DMH1 (Figure 3D). The MAF/MAFB and ZNF750 transcription factor axis drives the epidermal terminal differentiation transcription program (Lopez-Pajares et al., 2015). These results imply that activation of BMP signaling functions upstream of this axis.

**Figure 4:**
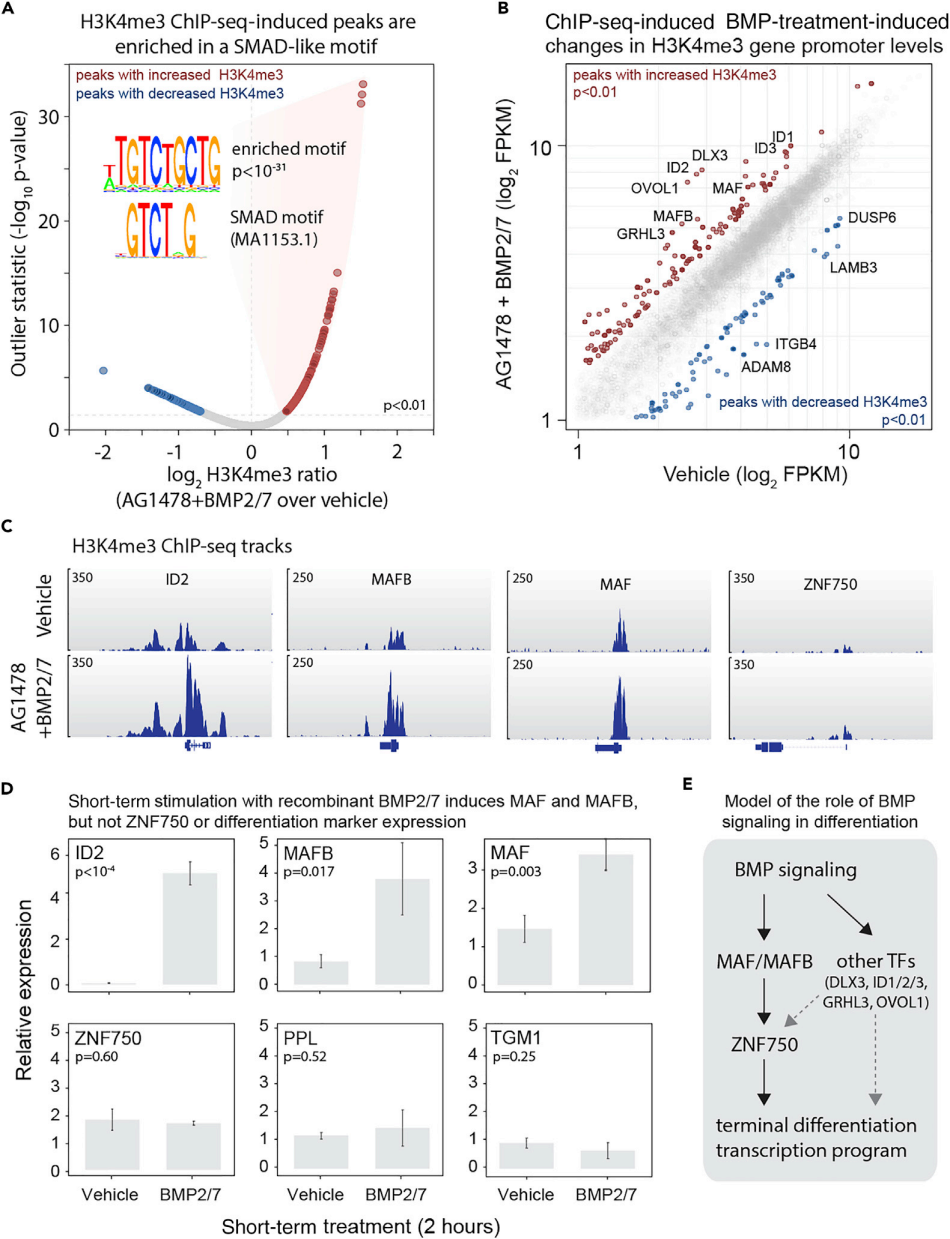
The Terminal Differentiation Transcription Factors MAF/MAFB Are Downstream Targets of the BMP Pathway (A) ChIP-sequencing of the active gene mark H3K4me3 identifies potential downstream targets after BMP stimulation. Scatterplot of normalized, log-transformed H3K4me3 ChIP-seq signals from cells incubated with and without recombinant BMP2/7 in the context of EGFR inhibition. Genes with increased or decreased H3K4me3 signals (p < 0.01, outlier statistics) at their transcription start site are highlighted in red and blue, respectively. Examples of relevant activated and inhibited genes are indicated. (B) Gene promoters of genes with induced H3K4me3 levels are enriched in an SMAD-like motif. The sequences (TSS ± 2.5 kb) of the genes with induced H3K4me3 levels were subjected to a *de novo* motif enrichment search. One of the enriched motifs showed high similarity to a known SMAD motif. (C) H3K4me3 genome-browser tracks of ID2, MAF, MAFB, and ZNF750. (D) MAF and MAFB, but not ZNF750, are immediate-early responding genes downstream of BMP stimulation. RT-qPCR analysis of the indicated genes 2 hr after treatment with vehicle or recombinant BMP2/7 (n = 3, data represented as mean ± SD). (E) Model of the transcription regulatory network activated by BMP signaling during epidermal differentiation.

To identify potential transcriptional targets downstream of the BMP pathway, we performed chromatin immunoprecipitation followed by sequencing (ChIP-seq), using H3K4me3 ChIP-seq signals at the transcription start site (TSS) as a proxy for changes in transcriptional activity of a gene. We did not manage to obtain high-quality SMAD1 ChIP-seq profiles and reasoned that the changes in H3K4me3 signals might provide a first indication of downstream BMP signaling targets. Proliferating epidermal stem cells were treated with vehicle or recombinant BMP2/7 (in combination with AG1478) for 6 hr, after which they were harvested for ChIP-seq analysis. This identified 135 genes that showed a significant increase in H3K4me3 signal (FPKM, p < 0.01, outlier statistics) at their TSS, suggestive of increased transcriptional activity (Figures 4A and 4B). *De novo* motif discovery on the 2.5 kb up and downstream of these TSS regions revealed enrichment of an SMAD-like motif (p < 10^−31^, Figure 4A). This is in line with the notion that these genes are responsive to BMP signaling. This set included the classical BMP targets ID1, 2 and 3, demonstrating that our experimental approach identified known direct BMP pathway target genes (Figure 4B). In addition to this, the terminal differentiation regulating transcription factors GRHL3, MAF, and MAFB were among the top set of immediate BMP targets (Figures 4B and 4C). In contrast, the key MAF/MAFB target gene ZNF750 was not directly regulated downstream of the BMP pathway as determined by H3K4me3 ChIP-seq signal (Figure 4C). A limitation of this experiment is that we performed ChIP-seq analysis after 6 hr of treatment. Some of the observations may therefore reflect secondary effects of BMP treatment. In general, we would expect direct BMP target genes to show an immediate-early response to activation of the pathway. We tested this by treating proliferating keratinocytes with recombinant BMP2/7 for 2 hr followed by RT-qPCR analysis. This revealed that both MAF and MAFB mRNA levels were induced at this early time point, as was the classical BMP target gene ID2 (Figure 4D). In contrast, ZNF750 was not induced, nor were the differentiation markers periplakin (PPL) and TGM1. These results place BMP pathway activation immediately upstream of the MAF/MAFB transcription factors. These factors subsequently drive terminal differentiation programs through, among others, ZNF750 (Figure 4E, Lopez-Pajares et al., 2015).

## Discussion

We developed single-cell Immuno-Detection by sequencing (scID-seq) as a highly multiplexed single-cell (phospho-)proteomics approach based on our previously published ID-seq method. The combination of these two related techniques constitutes a toolbox spanning cell population-based drug screens to single-cell follow-up. This allows exploration of the heterogeneity of drug effectivity within a cell population, as well as effects on signaling and other cellular processes within individual cells after drug treatment. In principle, scID-seq can be applied to any biological system in which single cells can be obtained and high-quality antibodies are available. Moreover, scID-seq in combination with FACS-based sorting allows enrichment of specific and/or rare cell types *a priori*. Using this approach, we enriched for spontaneously differentiating cells based on their surface ITGB1 levels to study signaling pathway activity in individual human epidermal cells with distinct differentiation states. Measuring 69 (phospho-)proteins per cell demonstrated that, among others, BMP signaling is activated along the differentiation trajectory. Mechanistically, the BMP pathway stimulates the MAF/MAFB/ZNF750 axis to induce a transcriptional program during late-stage epidermal differentiation. Previous studies provided indications that stimulation with exogenous BMP ligands increases the expression of cell cycle inhibitory factors and select differentiation-associated genes, suggesting involvement of this pathway in human epidermal differentiation (Botchkarev, 2003, Fessing et al., 2010, Gosselet et al., 2007, Li et al., 2003, Yang et al., 2006). However, its timing and function during the differentiation process remained unclear. Using RNA sequencing and H3K4me3 ChIP-seq analysis in combination with inhibition and stimulation, we found that BMP signaling activation drives a terminal differentiation transcription program and the MAF/MAFB/ZNF750 transcription factor axis (Figures 3 and 4). Our findings have implications for our view on the progressing nature of keratinocyte differentiation. First, regional signaling pathway activity plays crucial roles in patterning and tissue specification during (early) development. Our findings implicate BMP pathway activation as an integral part of the transcription factor network stimulating epidermal differentiation. As the BMP ligand is produced and excreted by the cells into their local environment, our results provide a mechanistic explanation for coordinated expression program progression in a zonated fashion in a tissue context. Second, the identification of the cell intrinsic activation of the BMP pathway through upregulation of its ligand BMP2, in combination with our observation that the BMP pathways is responsible for the stimulation of a specific transcriptional program including late differentiation regulators (e.g., MAF/MAFB, DLX3 and OVOL2), suggests that this pathway is involved in a self-sustaining loop that keeps driving epidermal differentiation forward. Taken together, our results reveal a mechanistic role for BMP signaling in human epidermal differentiation and indicates that activation of (potentially autocrine) signaling loops enables cells to coordinate their transcriptional programs and ensure progressive differentiation.

### Limitations of the Study

Our work establishes that scID-seq has the potential to quantify many antibodies with high sensitivity in individual cells. As with any antibody-based approach, the quality of the antibodies is a limiting factor and should be defined carefully. Our current iteration of scID-seq, which includes FACS-based single-cell isolation, is limited in the number of cells that can be practically processed in parallel. A solution would be replacing the FACS and sample preparation with droplet-microfluidics, as in the Ab-seq approach, for instance (Shahi et al., 2017). With regards to our biological question, the data contains a comparatively low coverage of intermediate differentiation stages for the pseudo-timing analysis. As a consequence, the relative timing of the pathways that show dynamics during that stage cannot, currently, be temporally segregated at high resolution. Finally, the H3K4me3 ChIP-seq we performed is taken as a proxy for transcriptional changes and we cannot exclude some contribution of secondary or indirect responses that are not *de facto* immediately downstream of BMP activation. We therefore chose to include additional experiments to provide further indications of direct effects on the MAF/MAFB/ZNF750 axis.

## Methods

All methods can be found in the accompanying Transparent Methods supplemental file.

## Data and Software Availability

Sequencing data was deposited in the Gene Expression Omnibus (GEO) repository under series number GSE115926.

## Supplemental Information

Document S1. Transparent Methods and Figures S1–S7

Table S1. Overview of the Antibodies and DNA Barcodes Used in this Study, Related to Figure 1
