# Supplementary material for: Single-Cell ID-seq Reveals Dynamic BMP Pathway Activation Upstream of the MAF/MAFB-Program in Epidermal Differentiation
Source: iScience. 2018 Nov 9;9:412–22. doi: 10.1016/j.isci.2018.11.009 (PMC6249387; doi:10.1016/j.isci.2018.11.009)
Supplement: Document S1. Transparent Methods and Figures S1–S7 [file mmc1.pdf]

ISCI, Volume *9*

Supplemental Information

Single-Cell ID-seq Reveals Dynamic BMP Pathway Activation Upstream of the MAF/MAFB-Program in Epidermal Differentiation

Roderick A.P.M. van Eijl, Jessie A.G.L. van Buggenum, Sabine E.J. Tanis, Joost Hendriks, and Klaas W. Mulder

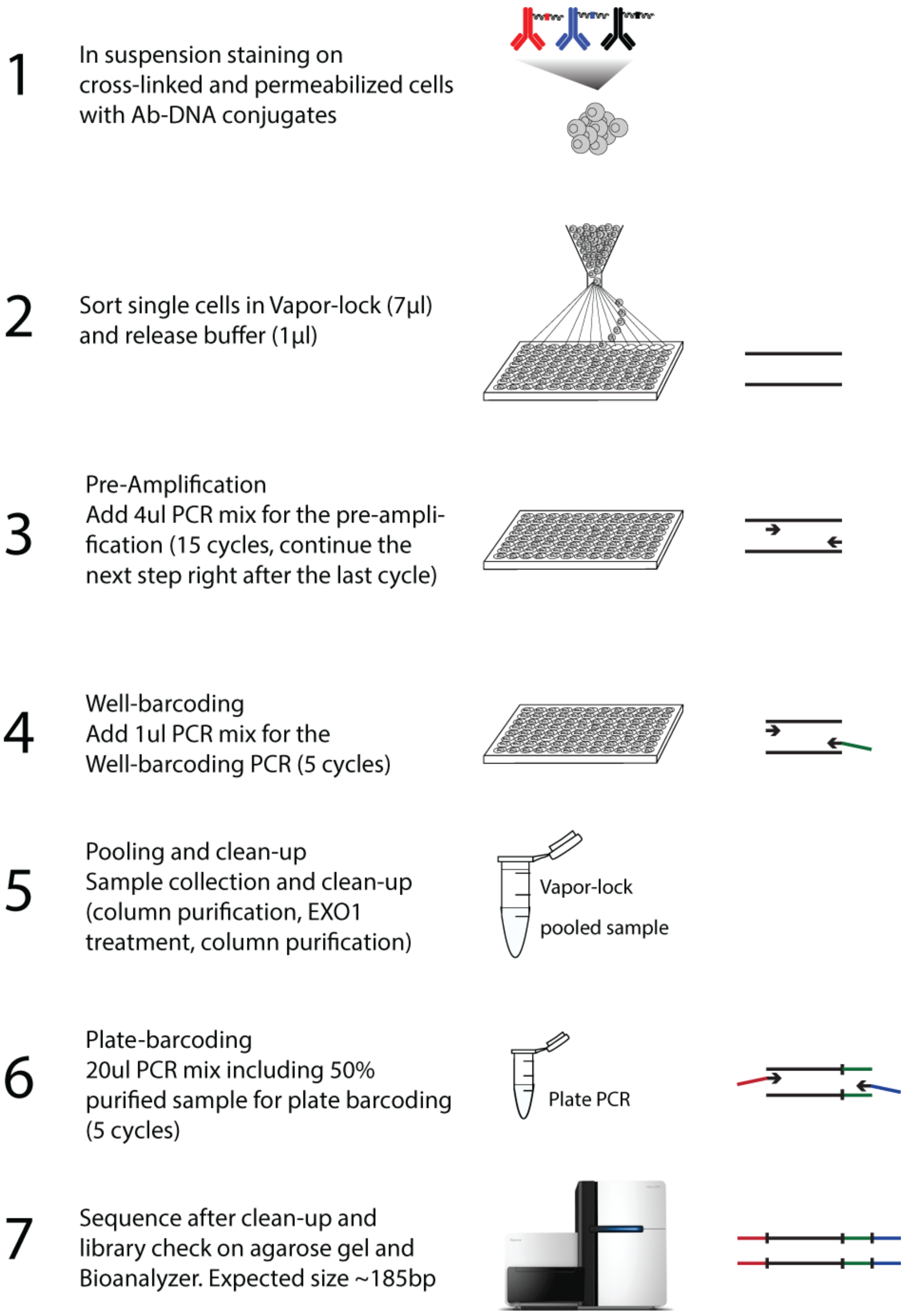

Figure S1: Schematic representation of the single-cell ID-seq work-flow. The scID-seq procedure entails crucial modifications compared to the original ID-seq protocol. First, cells are stained with the antibody-DNA conjugates in suspension, to allow single-cell sorting. Second, a pre-amplification step increased the yield and complexity of the single-cell libraries. This pre-amplification is done prior to adding a cell-specific barcode to the PCR products. From this point on, the library preparation is the same as described for ID-seq. Related to Figure 1.

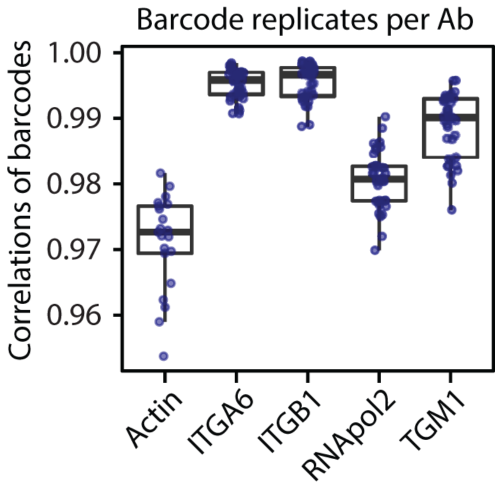

Figure S2: Nine technical replicates per individual cell highlight the reproducibility of scID-seq. Cells were stained with a mixture of 45 antibody-DNA conjugates consisting of 5 different antibodies that were each separately conjugated to 9 independent DNA-barcodes. The Pearson correlation among the 9 measurements for each antibody across individual cells indicates the reproducibility of scID-seq to quantify relative protein levels. Related to Figure 1.

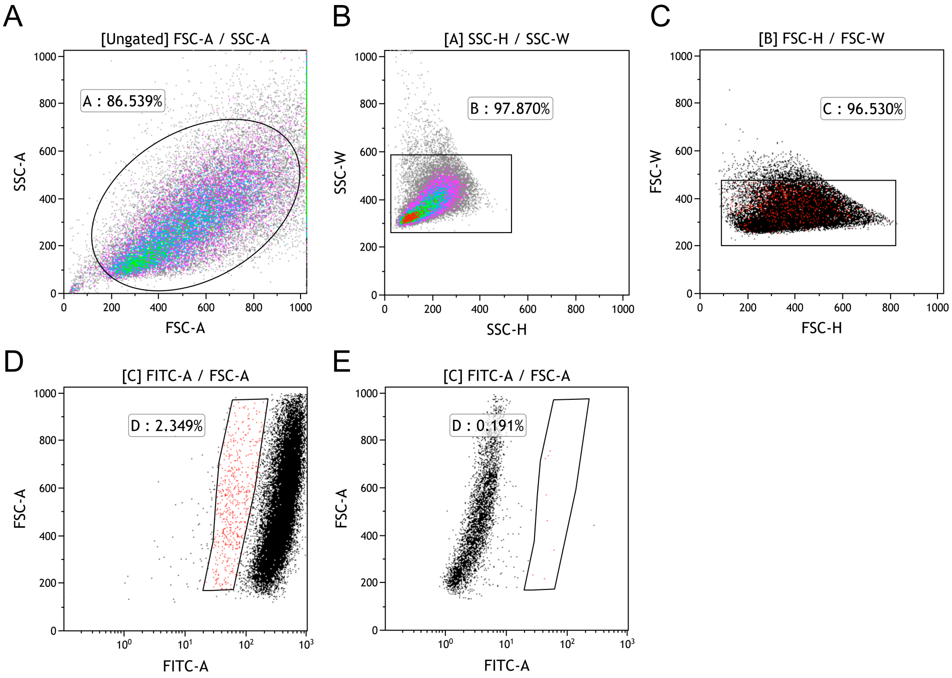

Figure S3: ITGB1 FACS strategy. (A-C) single cells were gated using forward and side-scatter characteristics. **(D)** Cells stained with low ITGB1-FITC levels were gated and sorted into individual wells (ITGB1low). ITGB1+ cells were selected from the total population. **(E)** Secondary antibody only stained cells indicate the specificity of the ITGB1-FITC staining. Related to Figure 2.

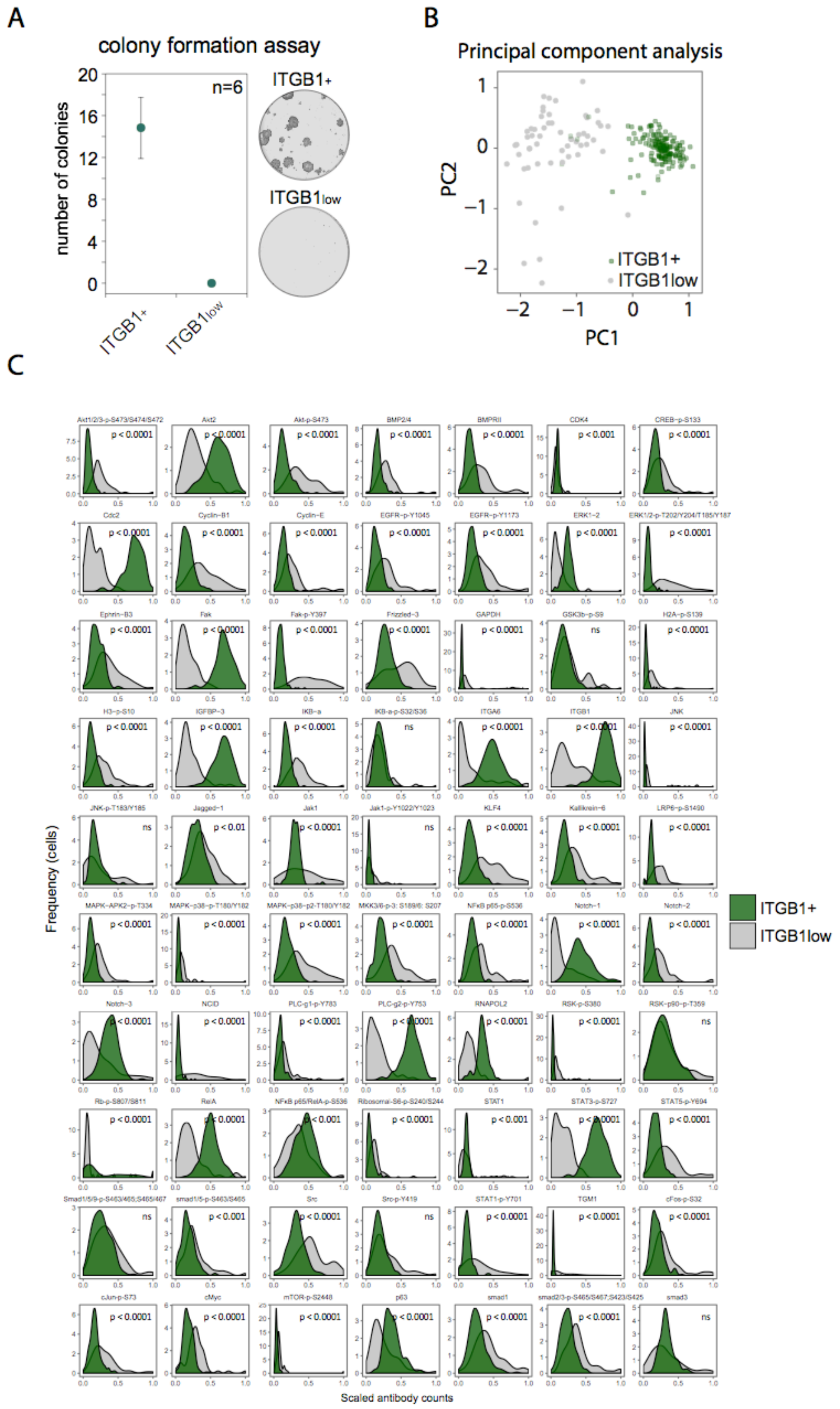

Figure S4: scID-seq on cells selected on ITGB1 level by FACS. (A) Cells with low ITGB1 surface levels represent differentiated keratinocytes. Cultured human keratinocytes were isolated based on their ITGB1 surface expression by FACS and subjected to colony formation assays. **(B)** ITGB1+ and ITGB1low cells display distinct scID-seq profiles. Human keratinocytes were subjected to staining with a panel of 70 antibody-DNA conjugates, isolated based on their

ITGB1 surface level by FACS and analyzed with scID-seq. Principal component analysis separated the ITGB1+ and ITGB1low populations. **(C)** Individual antibodies display dynamics between ITGB1+ and ITGB1low cell populations. Distributions of scaled signals of ITGB1+ and ITGB1low populations for 70 antibody-DNA conjugates. Statistically different distributions are indicated (Kolmogorov-Smirnov test, $p<0.001$). Related to Figure 2.

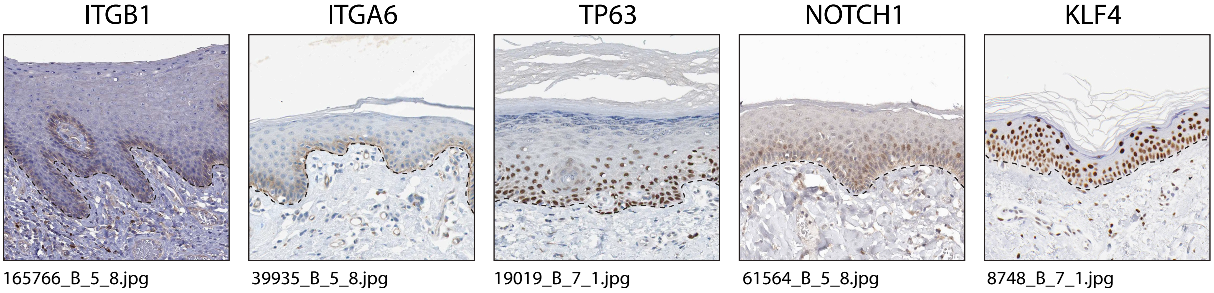

ITGB1 : basal layer & basement membrane staining, rapid decrease.
ITGA6 : basal layer & basement membrane staining, rapid decrease.
TP63 : basal layer staining, slow decrease.
NOTCH1 : throughout the epidermis, cell membrane staining in basal layer, the activated Notch Intracellular Domain localises to the nucleus in the suprabasal layers during differentiation.
KLF4 : increased expression in suprabasal layers during differentiation.

Dashed line indicates the separation of the dermis and the epidermis by the basement membrane.

Figure S5: Dynamics of known markers used to derive the pseudo-time line as assessed by immunohistochemical staining of human epidermis. Publicly available IHC staining of validated antibodies against the indicated epidermal marker proteins. Data from the human protein atlas. Related to Figure 2.

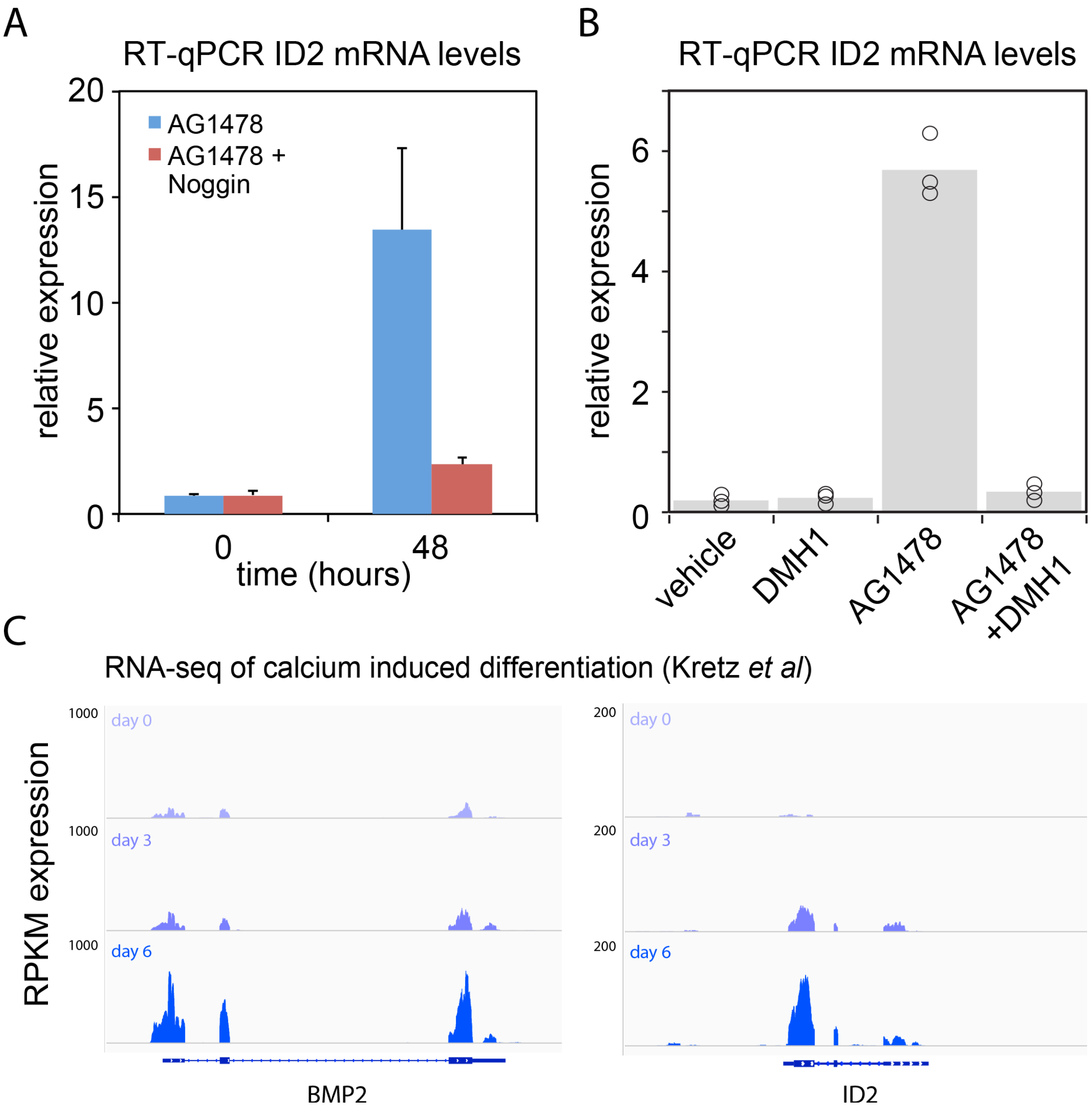

Figure S6: The BMP pathway target gene ID2 is induced during differentiation in a BMP-BMPR interaction dependent manner. (A) Human keratinocytes were induced to differentiate with the EGFR inhibitor AG1478 in the presence or absence of recombinant noggin for 48 hours and subjected to RT-qPCR analysis of the ID2 gene. **(B)** BMP2 and ID2 mRNA expression is induced during a time-course of calcium induced differentiation. Related to Figure 3.

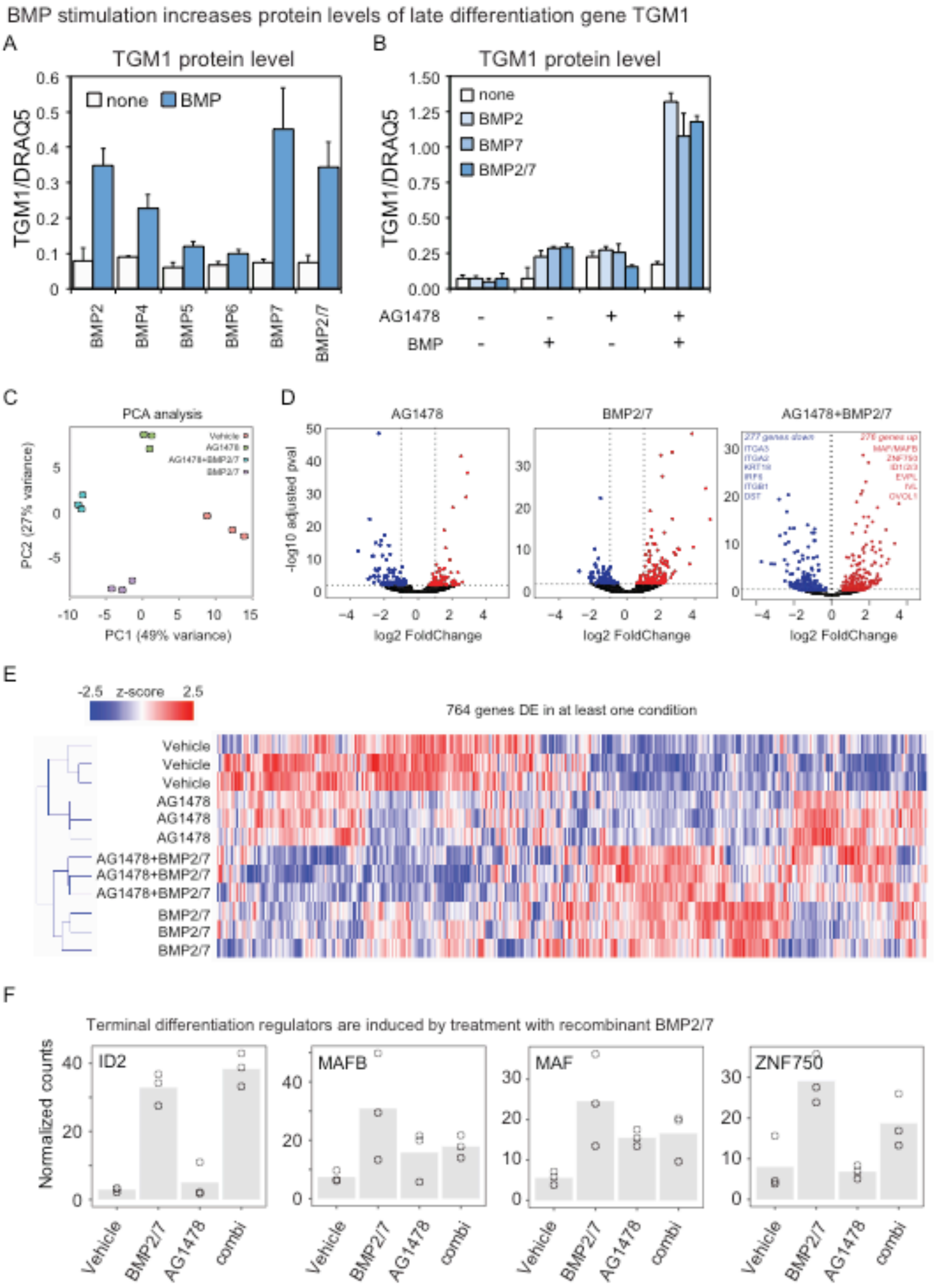

Figure S7: Stimulation with recombinant BMPs induces differentiation gene expression. (A) Recombinant BMPs stimulate endogenous TGM1 protein expression. Cells were stimulated with the indicated recombinant BMP proteins for 48 hours and subjected to In-Cell-Western analysis using TGM1 specific antibodies. Measurements were corrected for cell density using the DNA staining agent DRAQ5. n=3 -/+ SD **(B)** Recombinant BMPs and AG1478 syngergise to stimulate endogenous TGM1 protein expression. Cells were stimulated with the indicated recombinant BMP proteins for 48 hours in combination with the EGFR inhibitor AG1478 and subjected to In-Cell-Western analysis using TGM1 specific antibodies. Measurements were corrected for cell density using the DNA staining agent DRAQ5. n=3 -/+ SD**. (C)** Recombinant BMPs, AG1478 and the combined treatment lead to distinct transcriptional responses. Cells

were stimulated with the indicated treatments for 48 hours and subjected to RNA-sequencing. Principal component analysis of differentially expressed genes indicates condition specific transcriptional effects. **(D)** Identification of differentially expressed genes per condition. Volcano plots indicate the log2 fold-change of mRNA expression between vehicle and the indicated condition on the x-axis. The y-axis represents the -log10 transformed p-value (FDR corrected t-test). **(E)** Hierarchical clustering highlights condition specific differences in transcriptional responses. Heatmap of differentially expressed genes (z-score normalised across the samples) clustered on Pearson's correlation with average linkage for both genes and samples. **(F)** 48 hours treatment with recombinant BMPs stimulates expression of transcription factors involved in late epidermal differentiation. Related to Figure 3.

Transparent methods

Cell culture and reagents

Primary pooled human epidermal stem cells derived from foreskin were obtained from Lonza. Cells were cultured and expanded as previously reported (Gandarillas and Watt, 1997). Briefly, cells were cultured on a feeder layer of J2-3T3 cells in FAD medium (Ham's F12 medium/Dulbecco's modified Eagle medium (DMEM) (1:3) supplemented with 10% batch tested fetal calf serum (FCS) and a cocktail of 0.5 µg/ml of hydrocortisone, 5 µg/ml of insulin, 0.1 nM cholera enterotoxin, and 10 ng/ml of epidermal growth factor) supplemented with Rock inhibitor (Y-27632, 10 µM). J2-3T3 cells were cultured in DMEM containing 10% bovine serum and inactivated with Mitomycin C (SCBT) upon seeding the epidermal stem cells. For experiments epidermal stem cells were transferred to Keratinocyte Serum Free Medium (KSFM) supplemented with 0.2 ng/ml Epidermal Growth Factor and 30 µg/ml bovine pituitary extract from Life Technology until 70% confluent. Cells were treated with AG1478 (10 µM, Calbiochem), DMH-1 (1 µM, RND systems) or BMP2/7 (200 ng/ml, R&D systems). All media were supplemented with 1% penicillin/streptomycin antibiotics.

Antibody conjugation with dsDNA barcodes

Antibodies and dsDNA were functionalized and conjugated as described (van Buggenum et al., 2016). Antibody details are provided in supplemental Table 1. In short, antibodies were functionalized with NHS-s-s-PEG4-tetrazine (Jena Bioscience) in a ratio of 1:10 in 50 mM borate buffered Saline pH 8.4 (150 mM NaCl). Then, N3-dsDNA was produced and functionalized with DBCO-PEG12-TCO (Jena Bioscience) in a ratio of 1:25 (oligo list). Finally, purified functionalized antibodies were conjugated to purified functionalized DNA by 4-hour incubation at room temperature in borate buffered saline pH 8.4 in a ratio of 4:1 respectively. The reaction was quenched with an excess of 3,6-diphenyl tetrazine. The conjugation efficiency and quality were checked on an agarose gel, confirming that a substantial amount of DNA conjugated with the antibody. Ultimately, conjugates were equally pooled for staining's in scID-seq.

Immunostaining and single-cell sorting

Cells (> 3 x 10^6) were harvested with trypsin and cross-linked in suspension by incubating for 10 minutes with 4% paraformaldehyde (PFA) in PBS following a quenching step of 5 minutes with 125 mM Glycine in PBS. Removal of PFA and Glycine occurred through washing twice with wash buffer (0.1x Pierce™ Protein-Free Blocking Buffer from Thermo in PBS). Then, cells were blocked in 500µl blocking buffer (0.5x 0.1x Pierce™ Protein-Free Blocking Buffer, 200 µg/ml boiled salmon sperm DNA, 0.1% Triton-X 100, in PBS) at room temperature for 30-60 min. Staining with the conjugate mix occurred overnight at 4°C in 500 µl blocking buffer and pre-staining's were performed at room temperature for 1-2 hours. After each staining, cells were washed 3x in 5ml wash buffer. Cells were sorted single cell with the BD FACSAria SORP flow cytometer (BD biosciences) in 96 well PCR plates containing 1µl release buffer (10 mM DTT in 15mM Tris, pH 8.8) and 7µl Vapor-lock (Qiagen). For selection of ITGB1 negative cells, a primary and secondary pre-stain was done with 2.5 µg/ml anti-ITGB1 (P4D1) and 1:1000 Alexa488 goat anti-mouse (Life technology, 1484573). Plates were stored at -20°C until use.

Barcoding and library preparation for next generation sequencing

For the library preparation 3 PCR steps were performed to amplify the antibody barcodes and to add barcodes specific for the well and the plate of each cell. The barcoding occurred with

the same sequences used in ID-seq (van Buggenum et al., 2018). For the first PCR step 15 cycles were run after adding to each well a 4 µl reaction mix containing the Herculase II Fusion DNA Polymerase (Agilent), dNTPs, 5x Herculase buffer and 0.1µM amplification primers (Forward 5'-CACGACGCTCTTCCGATCT-3', Reverse 5'- TCGCTTATCTGTTGACTGAT-3'). Directly after the first PCR step, 5 extra cycles were run after adding 1 µl mix containing Herculase buffer 0.2 µM forward amplification primer and 0.2 µM reverse well barcoding primer. Then all material was pooled per plate, Vapor-lock was removed and a clean-up was performed with the QIAquick PCR Purification Kit, an EXO1 treatment to degrade remaining primers followed by another purification. Another 5 cycles were run in PCR 3 with a 20 µl reaction containing pooled and purified plate sample and 0.1 µM plate barcoding primers (Fw_long 5'-AATGATACGGCGACCACCGAGATCTACACTCTTTCCCTACACGACGC TCTTCCGATCT -3' and specific plate reverse). After repeating the clean-up, the libraries were checked on agarose gel and with the Bioanalyzer (Agilent) to confirm the size of the DNA fragments (expected size around 185 bp).

scID-seq data analysis

Sequence data from the NextSeq500 (Illumina) was demultiplexed using bcl2fastq software (Illumina). Then, all reads were processed using our dedicated R-package (van Buggenum et al., 2018). In short, the sequencing reads were split using a common "anchor sequence" identifying the position of the UMI sequence, Barcode 1 (antibody specific) and Barcode 2 (well specific) sequence. After removing all duplicate reads, the number of UMI sequences were counted per barcode 1 and 2. Finally, barcode 1 ("antibody)" and barcode 2 ('well') sequences were matched to the corresponding. For scID-seq, a threshold was set based on the total UMI count per well difference between high- and low-quality cells or empty wells. Antibodies with a median of <10 counts per cell were removed from the dataset. Cells that displayed strong outliers for a single antibody (cut-off > 5x standard deviation of the mean of the population) were removed from the analysis. After this, cells were normalized through subsampling and antibody counts were normalized on a scale from 0 to 1 using the formula (x-min(x))/(max(x)-min(x)). Principal component analysis was done with the R-package "pcaMethods". Additional statistical analysis and visualization of the data was done in R and Excel.

Colony formation assays

Epidermal stem cells were sparsely seeded on a feeder layer in a 6 well plate (500 cells/well) and cultured for at least 7 days to form colonies. After colony formation, cells were washed with PBS and fixed by incubating 10 minutes with 4% paraformaldehyde (PFA) in PBS. PFA removal occurred with 3 PBS washes. For the imaging of the colonies, a DNA stain was performed with DRAQ5 (1:4000, Biostatus) for 1 hour at room temperature. After 3 PBS washes, plates were scanned with the Odyssey system and the number of colonies quantified.

Immunostaining of human skin sections

Frozen sections of human foreskin were a kind gift from Prof. Fiona Watt and were obtained with informed consent and appropriate ethical review. Sections were fixed for 15 minutes with 4% paraformaldehyde, followed by 3 washes with PBS and permeabilisation with 0.2% triton-X100 in PBS for 10 minutes at RT. After blocking with 10% Bovine Serum in PBS for 1 hour, the sections were stained with antibodies against phosphorylated-SMAD1/5/9 (41D10, Cell Signaling Technologies) at 1:200 dilution in blocking buffer overnight at 4 degrees Celsius. Sections were washes 3x with PBS and stained with secondary antibodies (1:2000 dilution)

and DAPI (1:5000) for 90 minutes at RT. After mounting on a microscopy slide, images were acquired using a Leica IR laser confocal microscope.

RT-qPCR

Isolated RNA (Quicki-RNA TM MicroPrep, Zymo Research) was used for quantitative PCR analysis using iQTM SYBR Green Supermix with 20µM reaction volume, scanned on CFX-96 machine. Per gene, -2^Ct values were calculated and normalized to 18S RNA levels.

Transcriptome analysis with CEL-seq2

Isolated RNA (Qucki-RNA TM MicroPrep, Zymo Research) was used for transcriptome analysis via a slightly modified CEL-seq2 procedure (Hashimshony et al., 2016). See Table 1 primer sequences. In short, 100 pg purified RNA was used in 2 µl reverse transcription reactions containing Maxima H minus reverse transcriptase (ThermoFisher). The reactions were covered with Vapor-Lock (7 µl, Qiagen). Different primer sequences were designed and used (Table 1), allowing 63 nt long read 1 of mRNA, and 14 nt long read 2 with the sample barcode and UMI. NextSeq500 (Illumina) was used for sequencing.

Table 1. Primer sequences used during CEL-seq2 procedure.

Reverse transcription primer	5’GCCGGTAATACGACTCACTATAGGGGTTCAGACGTGTGCTCTTCCGATCTNNNNNNNN[6ntsamplebarcode]TTTTTTTTT TTTTTTTTTTTTTV3’
random-octamer-primer for reverse transcription of amplified RNA	5’CACGACGCTCTTCCGATCTNNNNNNNN3’
library PCR Primers	5’AATGATACGGCGACCACCGAGATCTACA CTCTTTCCCTACACGACGCTCTTCCGATCT3’<br>5’CAAGCAGAAGACGGCATACGAGAT[6ntindex]GTGACTGGAGTTCAGACGTGTGCTCTTCCGATC3’

CEL-seq2 data analysis

CEL-seq2 sequencing data was processed using as described (Hashimshony et al., 2016). In brief, high quality reads were filtered, and used for mapping. In brief, the count matrix was loaded as Seurat object, and used to visualize number of total UMI counts, number of genes and number of mitochondrial genes per sample. One out of three vehicle controls in the DMH-1 experiment did not pass the QC checks (with 50% less reads/sample) and was removed from further analyses. The count matrix was loaded into DESEq2 data object, to allow easy normalization, filtering, PCA and differential expression analysis. For analysis of the CEL-seq2 data, the DESeq2 R-package (Love et al., 2014) was used to normalize UMI counts, perform PCA analysis and determine differential expressed genes. Gene Ontology overrepresentation analysis was performed using the GeneTrail webtool (Backes et al., 2007)

ChIP-sequencing

Cells in KSFM ($2.5x10^6$) were incubated with vehicle/DMSO or BMP2/7+AG1478 for 6 hours. After harvesting by trypsinisation, the cells were cross-linked with 1% formaldehyde in PBS for 10 minutes, quenched for 5 minutes with 125 mM glycine in PBS and washed in PBS at 4

degrees Celsius. Cross-linked cells were incubated 4 hours in lysis buffer (5 mM Tris-HCl pH 8.0, 85mM NaCl, 0.5% NP40, 1X PIC) on ice, following 50 strokes with a dounce homogenizer to enrich for nuclei. Nuclei were sonicated in sonication buffer (50 mM Tris-HCl pH 8.0, 10 mM EDTA, 0.1% SDS, 150mM NaCl and 0.5% deoxycholic acid) to get an average chromatin fragment length of 500 bp. Chromatin extracts were incubated with 1 µg H3K4me3 antibody (ab8580, AbCam) overnight. Antibodies were captured for 4 hours with 100 µl protein G-coated magnetic beads (Life Technology). Subsequently, beads were washed 5X in RIPA buffer. The retrieved chromatin was reverse cross-linked overnight at 65°C followed by a 1-hour incubation step with proteinase K (1µg/µl) and RNAse A (1µg/µl) at 37°C. The DNA was purified with the Qiaquick PCR purification kit. Using the KAPA Biosystems kit (#KK8504), between 0.5 and 5ng ChIP-derived DNA was trimmed, A-tailed, provided with Nextflex adaptors and amplified with 10 PCR cycles. Subsequently, DNA was size-selected with the E-Gel® iBase™ Power System (Invitrogen) to purify for fragments between 300 and 400bp. The libraries were quantified on the Agilent 2100 Bioanalyzer and evaluated by qPCR to confirm representation of enrichments at specific loci. The libraries were sequenced with the Nextseq500. Reads were quality checked and aligned to the human hg19 genome with the Burrows-Wheeler Alignment tool (BWA) and processed with SAMtools to generate BAM files. Peaks were called from BAM files using "Model-based analysis of ChIP-Seq version 1.4" (MACS14) with a p-value cut-off of $1x10^{-8}$. The ratio of the fragments per kilobase per million reads (FPKM) values of the called H3K4me3 peaks were z-score transformed and subjected to outlier statistics assuming a normal distribution. De novo motif discovery was performed with the Trawler software (Dang et al., 2018). The enriched motifs were compared to known motifs in the JASPAR database using PWMtools (http://ccg.vital-it.ch/pwmtools).

Data and Software Availability

All sequencing data are available from GEO under series number GSE115926.
